# Structural Basis and Inhibitor Development of SARS-CoV-2 Papain-like Protease

**DOI:** 10.3390/molecules31030474

**Published:** 2026-01-29

**Authors:** Junshuai Wang, Yuancong Xu, Yishu Yang, Botao Zhang, Sixu Chen, Zhaoyang Li, Haojia Zhu, Huai Yang, Hongtao Wang, Yubai Zhou, Peng Cao, Baiqiang Zhai, Yong Gong

**Affiliations:** 1 College of Chemistry and Life Science, Beijing University of Technology, Beijing 100124, China; wjunshuai@emails.bjut.edu.cn (J.W.); xuyuancong@bjut.edu.cn (Y.X.); yishu-y@bjut.edu.cn (Y.Y.); zhangbotao@emails.bjut.edu.cn (B.Z.); sixuchen@emails.bjut.edu.cn (S.C.); zhaoyangli@emails.bjut.edu.cn (Z.L.); zhj2023@emails.bjut.edu.cn (H.Z.); yanghuai@emails.bjut.edu.cn (H.Y.); zhouyubai@bjut.edu.cn (Y.Z.); pengcao@bjut.edu.cn (P.C.); 2 Beijing Synchrotron Radiation Facility, Institute of High Energy Physics, Chinese Academy of Sciences, Beijing 100049, China; 3 Railway Food Safety Management Engineering Technology Research Center, Zhengzhou Railway Vocational & Technology College, Zhengzhou 451400, China; 10554@zzrvtc.edu.cn; 4 Institute of Matter Science, Beijing University of Technology, Beijing 100124, China

**Keywords:** SARS-CoV-2, papain-like protease, crystal structure, drug discovery

## Abstract

Papain-like protease (PLpro), a crucial functional domain of the SARS-CoV-2 non-structural protein 3 (nsp3), plays a dual role in both hydrolyzing viral polyprotein precursors and modulating host immune responses. These critical functions position PLpro as a key target in the ongoing development of antiviral therapies for SARS-CoV-2. This review analyzes more than 100 PLpro-ligand co-crystal structures and summarizes the major binding modes between these ligands and PLpro. Most of these ligands bind to sites analogous to those targeted by the classical non-covalent inhibitor GRL0617, primarily involving the P3 and P4 subsites and the BL2 loop. Based on these structural insights, optimized inhibitors have expanded targeting beyond the canonical binding site to auxiliary regions such as the BL2 groove and the Val70 site, and in some cases toward the catalytic Cys111 buried within a narrow pocket. Certain ligands identified through various screening approaches bind to non-canonical or allosteric regions, such as the S1 and S2 sites or the zinc-finger domain, engaging PLpro through distinct interaction modes and thereby offering additional opportunities for PLpro inhibitor design. The review also discusses potential strategies for future PLpro inhibitor development informed by recent structural advances. Taken together, these structural and functional insights support ongoing efforts in the structure-guided design and optimization of PLpro inhibitors.

## 1. Introduction

**Figure 1:**
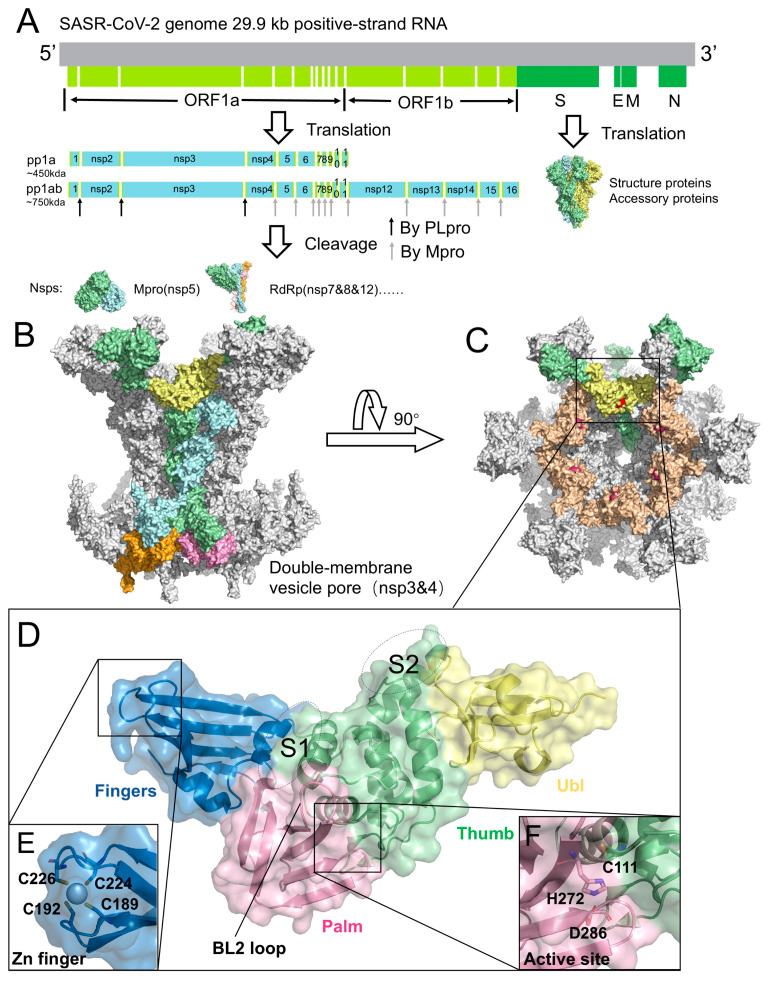
Genome, replication organelles, and structural features of SARS-CoV-2 PLpro. (**A**) Genome organization of SARS-CoV-2. ORF1a and ORF1b are translated into polyproteins pp1a and pp1ab, which are cleaved by PLpro and Mpro into individual nsps. Representative structures: Mpro (nsp5, PDB code: 7MB3), RdRp (nsp7/8/12, PDB code: 6YYT), and Spike protein (PDB code: 6VYB). (**B**) Architecture of the DMV pore complex, composed of six long nsp3 (green), six short nsp3 (blue), six long nsp4 (pink), and six short nsp4 (orange). The PLpro domain of long-chain nsp3 is highlighted in yellow (PDB code: 8YAX). (**C**) Top view of the DMV pore complex. PLpro, located on the cytosolic surface, is shown in yellow and wheat colors, with the catalytic residues highlighted in red. (**D**) Structure of PLpro (PDB code: 6WZU), illustrating the Ubl domain (yellow), Thumb domain (green), Finger domain (blue), and Palm domain (pink). The S1 and S2 sites are labeled. (**E**) Zinc-finger motif of PLpro. (**F**) Catalytic triad of PLpro, consisting of Cys111, His272, and Asp286.

Coronaviruses (CoVs) were first identified in humans in the 1960s and include highly pathogenic strains such as SARS-CoV-2 (2019), SARS-CoV (2003), and MERS-CoV (2012), as well as less pathogenic strains such as HKU1, OC43, NL63, and 229E [1,2]. Among these, SARS-CoV-2 has exerted the greatest global impact, owing to rapid viral evolution and the emergence of variants that have complicated both prevention and treatment [3,4,5]. SARS-CoV-2 is an enveloped virus with a positive-sense, single-stranded RNA genome of approximately 29.9 kb (Figure 1A) [6,7]. At the whole-genome level, it shares ~82% nucleotide sequence identity with SARS-CoV, whereas similarity to MERS-CoV is substantially lower (~56.9%), and ~50–52% with HCoV-OC43 and HCoV-HKU1 [8,9,10,11,12].

The SARS-CoV-2 genome encodes 29 viral proteins (Figure 1A). Its replication strategy relies on the translation of two large polyproteins, pp1a and pp1ab, which are subsequently cleaved into 11 or 16 non-structural proteins (nsps) that direct viral RNA synthesis, transcription, and virion assembly [13,14,15,16,17]. This proteolytic processing is mediated by two cysteine proteases: the main protease (Mpro, 3CLpro) and the papain-like protease (PLpro), both of which are indispensable for completion of the viral life cycle [18,19,20]. Mpro recognizes the cleavage sites defined by the LQ↓A/S motif, whereas PLpro targets the LXGG↓XX motif and cleaves the nsp1/2, nsp2/3, and nsp3/4 junctions [21,22,23]. In addition to polyprotein maturation, PLpro also counteracts host innate immune responses by removing ubiquitin and ISG15 conjugates from cellular proteins, thereby promoting viral immune evasion [24,25,26,27,28,29,30,31,32].

Both Mpro and PLpro are established as pivotal targets for antiviral drug development [33,34,35,36,37]. Several Mpro inhibitors have progressed to clinical use, including nirmatrelvir (Paxlovid, Pfizer, 2022) [38,39] and leritrelvir (Lerelinq, Zhongsheng Ruichuang Biotechnology, 2023) [40]. Despite these advances, Mpro-targeted therapies face ongoing challenges, including the emergence of resistant variants and pharmacokinetic constraints [41,42]. In vitro selection and serial passaging using Mpro variants have identified multiple inhibitor-selected Mpro substitutions, including S144A, A173V, and F305L. These substitutions reduce susceptibility by reshaping local active-site interactions or altering conformational dynamics [43,44]. In addition to the risk of resistance, pharmacokinetic and clinical limitations, including treatment regimen constraints and potential drug interactions, pose significant barriers to the long-term effectiveness of Mpro-based monotherapy [43].

These considerations emphasize the importance of diversifying antiviral targets beyond Mpro. In this context, PLpro provides an orthogonal antiviral strategy. Mechanistically distinct from Mpro, PLpro functions as a deubiquitinase/deISGylase, suppressing host innate immune signaling [31,32]. Consequently, inhibiting PLpro may both impair viral replication and help restore host antiviral responses. To date, more than 100 high-resolution crystal and co-crystal structures of SARS-CoV-2 PLpro have been reported (crystallographic data summarized in Supplementary Table S1). These structures shed light on its substrate-recognition features and provide a solid framework for developing PLpro inhibitors.

In this review, our goal is to provide a comprehensive, structure-centered overview of SARS-CoV-2 PLpro inhibition and to extract key design principles that can guide the rational development of next-generation inhibitors. Available X-ray co-crystal structures of PLpro-ligand complexes are systematically summarized, with emphasis on how distinct inhibitor classes engage the enzyme and modulate its activity. Representative compounds with resolved binding poses are highlighted, and key limitations, including potency, selectivity, and drug-like properties, are discussed alongside practical directions for optimization. Reported inhibitors lacking structural validation are also briefly surveyed to provide a more complete view of the field. Finally, emerging opportunities that integrate structural biology with new discovery strategies are outlined to help accelerate the development of PLpro-targeted antivirals.

## 2. Dual Biological Function of PLpro in Viral Replication and Immune Evasion

PLpro is a functional domain of nsp3 encoded within the viral polyproteins pp1a and pp1ab [45]. SARS-CoV-2 nsp3 is a 1945-residue multidomain transmembrane protein that cooperates with nsp4 to remodel intracellular membranes and form dou-ble-membrane vesicles (DMVs), which serve as replication platforms for viral RNA synthesis [46,47]. Recent cryo-electron tomography resolved the nsp3-nsp4 pore com-plex at ~4.2 Å resolution, revealing a 12-subunit channel traversing the DMV mem-brane, with PLpro extending into the cytosol (Figure 1B,C) [48]. Within nsp3, PLpro is generally delineated as a 315-residue catalytic core (Glu746-Lys1060) located on the cytosolic surface of the pore (Figure 1D,E). This region harbors the catalytic cleft and constitutes the proteolytic module responsible for cleavage at the nsp1/2, nsp2/3, and nsp3/4 junctions [49,50]. Structurally, PLpro adopts a papain-like cysteine protease fold characterized by a conserved right-hand architecture (finger-palm-thumb) and a Cys-His-Asp catalytic triad (Figure 1E), closely resembling papain. It also contains dis-tinctive elements, including an N-terminal ubiquitin-like (Ubl) domain and the BL2 loop, which contribute to substrate recognition and regulation beyond those of canon-ical papain-like proteases (Figure 1D).

Beyond its role in polyprotein processing, PLpro exerts broad immunomodulatory effects through deubiquitinating and deISGylating activities. Removal of ubiquitin from host proteins perturbs proteasomal degradation and ubiquitin-dependent signaling, stabilizing host and viral factors that promote viral replication [24]. In parallel, hydrolysis of ISG15 conjugates suppresses type I interferon production and attenuates NF-*κ*B signaling, thereby weakening innate antiviral responses [25,26,27,28,29,30]. Notably, SARS-CoV-2 PLpro exhibits a stronger preference for deISGylation compared to its SARS-CoV ortholog. It binds human ISG15 and K48-linked di-ubiquitin with nanomolar affinity through a dual-domain recognition mechanism, providing a structural basis for its potent antagonism of interferon signaling [32]. Furthermore, PLpro removes K63-linked ubiquitin from immune sensors such as STING (e.g., at Lys289), thereby disrupting the STING-IKK*ε*-IRF3 signaling axis, reducing IRF3 phosphorylation, and suppressing IFN-*β* induction [51]. Pharmacological PLpro inhibition has been shown to synergize with STING agonists, restoring type I interferon responses in airway epithelial cells [51].

## 3. Structural Analysis of the Catalytic Mechanism of PLpro

Structural studies, primarily based on X-ray crystallography, have been central to elucidating the molecular mechanism of SARS-CoV-2 PLpro. By providing atomic-level views of the active site and ligand-binding interfaces and capturing binding-induced conformational changes, these structures have delineated the residues and structural elements critical for catalysis and substrate recognition. Mechanistically, PLpro recognizes substrates via a restrictive P4-P1 mode within the catalytic cleft, with the S1 and S2 subsites and the flexible BL2 loop further shaping substrate specificity.

### 3.1. Substrate Recognition in the Catalytic Cleft

**Figure 2:**
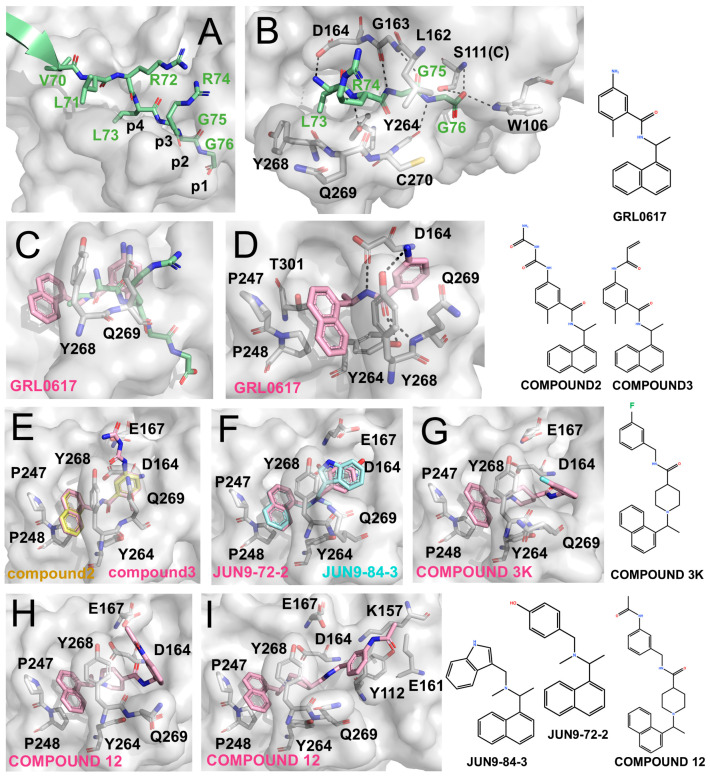
Structural development of PLpro inhibitors based on GRL0617 (The surface and carbon atoms of PLpro are shown in gray, ISG15 in green, and ligands in pink, cyan, or pale yellow). (**A**) Substrate recognition by PLpro. The LRGG motif of ISG15 binds at the active site (PDB code: 7RBS). (**B**) Detailed interactions shown in panel (**A**). (**C**,**D**) Two views of GRL0617 bound to the PLpro active site (PDB code: 7CJM). (**E**) Compounds **2** and **3** (PDB codes: 7JIR, 7JIT). (**F**) JUN9-72-2 and JUN9-84-3 (PDB codes: 7SDR, 7RZC). (**G**) Compound **3k** (PDB code: 7TZJ). (**H**,**I**) Two binding conformations of compound **12** (PDB code: 7E35).

The crystal structure of SARS-CoV-2 PLpro bound to human ISG15 revealed the molecular basis for recognition of the characteristic LXGG motif of the substrate [52]. As illustrated in Figure 2A, the leucine side chain at the P4 position of the ISG15 C-terminal tail is tightly buried in a hydrophobic pocket on PLpro defined by Asp164, Pro247, Pro248, Tyr264, Tyr268, and Tyr273, underscoring the strict requirement for leucine at this position. By contrast, the arginine at P3 of the substrate extends outward toward PLpro residues Gly161, Leu162, and Glu163. It forms a hydrogen bond with the backbone carbonyl of PLpro Leu162 but is otherwise largely solvent-exposed, reflecting PLpro’s relaxed specificity at this site and its mild preference for basic residues. These features are consistent with the viral and host cleavage motifs recognized by PLpro, including LNGG in nsp1/2, LKGG in nsp2/3, and LRGG in ubiquitin and ISG15 (Figure 2B). The strict requirement for glycine at both the P2 and P1 positions of the substrate can be explained by the narrow and elongated binding channels within PLpro, which sterically exclude bulkier residues. Substrate stabilization is further reinforced by backbone hydrogen bonds between PLpro and the substrate backbone, with additional contributions from the P3 arginine side chain. The BL2 loop makes a key contribution: Tyr268 projects into the P4 pocket to shield it from solvent, while Gln269 extends outward to help define the shape and polarity of the catalytic cleft (Figure 2B). Importantly, the intrinsic flexibility of the BL2 loop is crucial for both substrate binding and inhibitor engagement.

### 3.2. Auxiliary Binding Sites: S1 and S2 Sites

Beyond the catalytic cleft, additional substrate interactions at distal sites further refine PLpro specificity. Crystal structures of PLpro in complex with ubiquitin and ISG15 [25,32,52,53,54] have identified two key regions (Figure 1D): the S1 site, located at the thumb-finger junction (Arg166-Ala176), and the S2 site, positioned at the tip of the thumb domain adjacent to the Ubl region (Asp61-Tyr71). At the S1 site, Arg166, Glu167, and Tyr171 form multiple hydrogen bonds and electrostatic interactions with ubiquitin. Additionally, ubiquitin’s Trp123 inserts into the groove between Arg166 and Glu167, providing further stabilization. At the S2 site, PLpro Glu70 engages serine residues of ubiquitin through polar interactions. For ISG15 binding, Glu167 at the S1 site adopts a dual conformation and, together with Arg166, mediates extensive hydrogen bonding, whereas the S2 site contributes fewer stabilizing contacts. Computational studies further suggest that ISG15 associates with the S2 site via weaker, non-ionic interactions, distinguishing it from ubiquitin binding [32].

## 4. Representative PLpro Inhibitors with Structural Insights

PLpro inhibitors have been discovered through structure-guided design/optimization and screening-based approaches including high-throughput and fragment-based screening. They are typically evaluated using in vitro assays (binding affinity, IC_50_, EC_50_, and CC_50_), together with co-crystal structures that define their binding modes. Structural studies of PLpro show that the catalytic cysteine (Cys111) is recessed within a narrow pocket, reducing accessibility for classical covalent inhibitors, such as E-64, N-ethylmaleimide, and iodoacetamide (Figure 2B) [33,37,55]. As a result, most reported compounds are non-covalent inhibitors, exemplified by GRL0617 and its derivatives, which exploit the P3 and P4 subsites. Aromatic scaffolds are commonly used to anchor the hydrophobic P4 pocket, while elongated substituents may extend toward Cys111, as in VIR250/251-derived analogues. Beyond the catalytic cleft, the BL2 groove adjacent to the active site accommodates allosteric ligands. The S1 and S2 subsites, which mediate recognition of ubiquitin and ISG15, provide opportunities to interfere with deubiquitinating and deISGylating functions. The zinc-finger motif represents another vulnerability, which can be disrupted by chelators or metal-based complexes that destabilize Zn^2+^ coordination, thus impairing enzyme function [56,57,58].

### 4.1. GRL0617 and Its Derivatives—Classical Prototype of Non-Covalent Inhibitors

GRL0617 is a landmark small-molecule, non-covalent competitive inhibitor of PLpro. Due to the high sequence conservation between SARS-CoV and SARS-CoV-2 PLpro (83% identity and 90% similarity) [49], GRL0617—originally identified through high-throughput screening and reported against SARS-CoV PLpro [50]—has served as a prototype scaffold for the development of SARS-CoV-2 PLpro inhibitors. Although GRL0617 exhibits only moderate potency, its distinctive binding mode has provided a foundational structural template that continues to guide optimization and scaffold diversification.

#### 4.1.1. GRL0617

GRL0617 exhibits an IC_50_ of ~2.1 μM against SARS-CoV-2 PLpro and an EC_50_ of ~1.4 μM in cell-based assays [59,60,61]. As shown by the co-crystal structure (Figure 2C,D), GRL0617 binds within the P3/P4 subsites: the naphthyl ring is anchored in the hydrophobic P4 pocket, while the adjacent methyl group occupies a deep hydrophobic cavity. The benzene ring is positioned at the P3 site, where its amino group forms hydrogen bonds with Tyr268 and Gln269 on the BL2 loop, and the linker region engages Asp and Tyr residues within the catalytic cleft.

#### 4.1.2. Direct Derivatives of GRL0617

Osipiuk et al. introduced long-chain derivatives (Compound **2** and Compound **3**; Figure 2E) that enhanced hydrogen bonding with Asp164 and Glu167 but caused displacement of the aromatic core, resulting in reduced potency (IC_50_ 5.7–6.4 μM) [62]. Similar trends were observed for other Snyder analogues (PLP_Snyder441, 494, 496, 608, and 630), demonstrating that preservation of the methyl-P4 hydrophobic interaction is critical for activity. Therefore, disruption of this interaction leads to a marked loss of inhibitory potency, highlighting the intrinsic limitations of direct scaffold elongation.

#### 4.1.3. Alternative Scaffolds Inspired by GRL0617

Jun Wang et al. developed the inhibitors JUN9-72-2 and JUN9-84-3, which retained the overall binding mode of GRL0617 but altered BL2-loop interactions by removing the linker carbonyl [63,64]. These modifications induced conformational changes in Tyr268 (Figure 2F) and resulted in improved potency (IC_50_ ~0.67 μM, EC_50_ ~7.93 μM for JUN9-72-2 and IC_50_ ~0.67 μM, EC_50_ ~17.07 μM for JUN9-84-3) [63,64]. Structural analyses suggest that such loop rearrangements may enable exploitation of an “open” BL2-loop conformation.

Subsequently, Shan et al. reported Compound **12** (IC_50_ ~2.0 μM), which incorporates three hydrophobic rings and binds in two distinct conformations across the PLpro dimer, highlighting its notable conformational plasticity (Figure 2H,I). Further optimization yielded Compound **19** with sub-micromolar potency (IC_50_ ~0.26 μM), although corresponding structural data have not yet been reported [65]. In parallel, Calleja et al. identified the compounds such as **3k** (IC_50_ ~1.5 μM) and 5c (IC_50_ ~0.7 μM) through high-throughput screening. These inhibitors feature multi-aromatic scaffolds with fluorine substitution that stabilize interactions with Tyr268 (Figure 2G) [66]. Together, these studies underscore the importance of multi-aromatic frameworks in stabilizing BL2 loop conformations and enhancing non-covalent inhibition of PLpro.

### 4.2. Expansion to Auxiliary Binding Sites near P3/P4

As additional PLpro binding modes have been elucidated, several subsites adjacent to the P3/P4 pocket have emerged as productive handles for improving inhibitor affinity. Notably, these include the BL2 groove, the Glu167 site, and the Val70 site.

#### 4.2.1. Expansion to the BL2 Groove as an Allosteric Target

**Figure 3:**
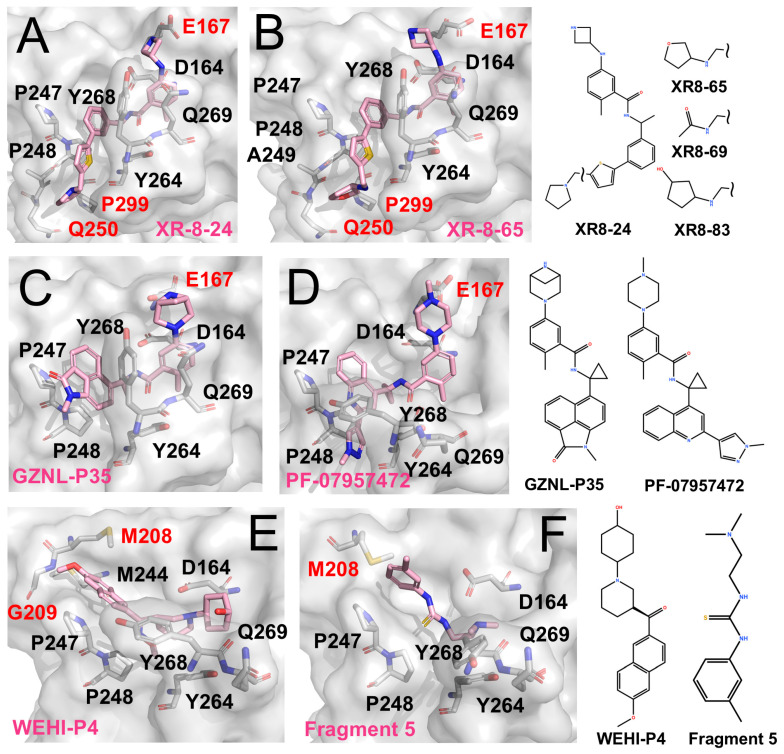
PLpro inhibitors engaging distinct auxiliary subsites (The surface and carbon atoms of PLpro are shown in gray and ligands in pink). (**A**,**B**) Two XR-series inhibitors occupying the active site and extending to the BL2 groove. (**C**) GZNL-P35 binding to the P3/P4 and Glu167 sites (PDB code: 8YX5). (**D**) PF-07957472 binding to PLpro C111S mutant (PDB code: 9CSY). (**E**) WEHI-P4 engaging the P3/P4 region and extending to the Val70 site (PDB code: 9CYD). (**F**) Fragment 5 binding to the P3/P4 region and the Val70 site (PDB code: 9BRV).

Shen et al. identified the BL2 groove adjacent to the active site as a promising pocket [67]. The BL2 groove is a narrow, elongated cleft that extends alongside the BL2 loop, spanning from the P4 hydrophobic pocket toward Gln250. They designed XR-8-24 (IC_50_ 0.56 μM, EC_50_ < 2 μM), which couples a biaryl core with a heterocyclic extension to engage this groove. Structural validation confirmed this binding mode and supported its affinity enhancement (Figure 3A,B) [67]. Notably, its analogues Compound **42** (IC_50_ ~0.81 μM) and Compound **10** (IC_50_ ~0.39 μM) showed pronounced antiviral effects in both cell-based and animal studies [68]. Building on this, Jun Wang et al. reported XR-8-derived inhibitors (e.g., JUN11243 and JUN11273) with sub-micromolar potency (IC_50_ ~0.5–0.7 μM; EC_50_ ~6 μM) [63].

#### 4.2.2. Glu167 as a Key Auxiliary Site

Glu167 is located near the P3 subsite, positioned outward from Asp164. Its carboxylate group can capture positively charged, extended side chains projecting from the P3 region, thereby strengthening local electrostatic and hydrogen-bonding interactions and improving overall affinity. This feature has been observed in XR-8-24, XR-8-65, and Compound **3**.

More recently, Lu et al. further optimized the scaffold, generating compounds such as GZNL-P35 (IC50 ~8.15 nM; Figure 3C) and P36 (IC50 ~6.45 nM, EC50 ~0.17 μM) with nanomolar potency [69]. These analogues replaced the naphthyl moiety with bulkier aromatics and introduced cyclopropyl substitutions. As a result, P4 binding was reinforced, and additional hy-drogen-bonding interactions with Glu167 were formed, leading to more than a 10-fold increase in potency. In parallel, Alpha A. Lee’s team performed several rounds of ma-chine-learning-guided optimization of GRL0617 and obtained PF-07957472 (EC50 ~147 nM; Figure 3D) [70]. Similarly to many GRL0617 derivatives, its large aromatic tail occupies the hydrophobic groove at the P4 site, while its slender head is oriented toward Glu167 to form specific polar contacts.

#### 4.2.3. Unexpected Discovery: The Val70 Site

JUN11313 was originally designed as a covalent inhibitor intended to target the BL2 groove. However, structural analysis showed that its thiophene ring, which was introduced to engage the BL2 groove, adopts an alternative orientation and instead packs near Met208 (Figure 3E) [71]. This region overlaps with the binding position of ubiquitin Val70 in the PLpro-ubiquitin complex, and the pocket was therefore termed the Val70 site.

Subsequently, Bader et al. identified the WEHI-P series through high-throughput screening followed by iterative optimization, yielding multiple potent inhibitors [72]. The series comprises compact, non-covalent scaffolds built around a central aromatic core with heterocyclic substituents, forming a relatively rigid framework that supports hydrophobic and π–π interactions while allowing peripheral groups to access adjacent auxiliary pockets. Compared with GRL0617-derived inhibitors, WEHI-P compounds show reduced reliance on BL2-loop engagement. A representative structural example is WEHI-P4 (IC_50_ ~19 nM; EC_50_ ~0.40 μM), which binds toward the upper region of the P4 site and extends into the Val70-associated pocket (Figure 3E), closely resembling the binding mode observed for JUN11313.

Taylor et al. employed an NMR-based fragment screening approach to identify several PLpro-binding fragments, among which fragments 5, 7, and 11 were further validated by crystallography [73]. Among them, Fragment 5 binds within the P3/P4 site and, in a distinct manner, anchors a benzyl group at the Val70 site (Figure 3F).

#### 4.2.4. Future Directions for Non-Covalent Inhibitors

Structural and functional studies clearly highlight the stratification among non-covalent chemotypes of PLpro inhibitors. GRL0617 serves as the foundational scaffold, with its P3/P4-centered binding mode and BL2-loop engagement providing a robust template for structure-guided optimization. Building on this foundation, targeted expansion into adjacent auxiliary subsites has led to the most consistent improvements. Specifically, extension into the BL2 groove (as exemplified by the XR-8 series) and engagement of Glu167 (as seen in the GZNL-P series and PF-07957472) have consistently resulted in submicromolar to nanomolar potency and, crucially, measurable antiviral efficacy in both cellular and animal models. Moreover, the identification of the Val70 site through compounds such as WEHI-P4 opens additional avenues for scaffold diversification. These series, therefore, represent some of the most mature and translationally promising non-covalent inhibitors reported to date.

Further gains in potency remain a key goal, and structure-guided optimization will likely continue to rely on the conformational plasticity of the BL2 loop to strengthen the interactions within the P3/P4 region. Equally important is improving selectivity. Because many GRL0617-inspired inhibitors target the vicinity of the catalytic cleft, potential off-target inhibition of host deubiquitinases remains a concern given the large family of related human enzymes. Rational modifications that extend into the BL2 groove or the Glu167 region may introduce more virus-specific contacts and thereby improve selectivity. Finally, the Val70 site appears to accommodate aromatic substituents, suggesting a recognizable auxiliary handle that could be leveraged to expand chemical space and improve inhibitor targeting capability.

### 4.3. VIRs and Peptide Inhibitors—Representative Covalent Inhibitors

VIR250 and VIR251 represent the first-generation covalent peptide-mimetic inhibitors of PLpro. Their LXGG-based peptide scaffolds demonstrate that covalent modification of Cys111 is an effective strategy, providing a complementary approach to the non-covalent inhibition. Building on this concept, later hybrid designs have combined electrophilic warheads with GRL0617-derived scaffolds, creating opportunities to improve potency and broaden chemical diversity.

#### 4.3.1. VIR250 and VIR251

**Figure 4:**
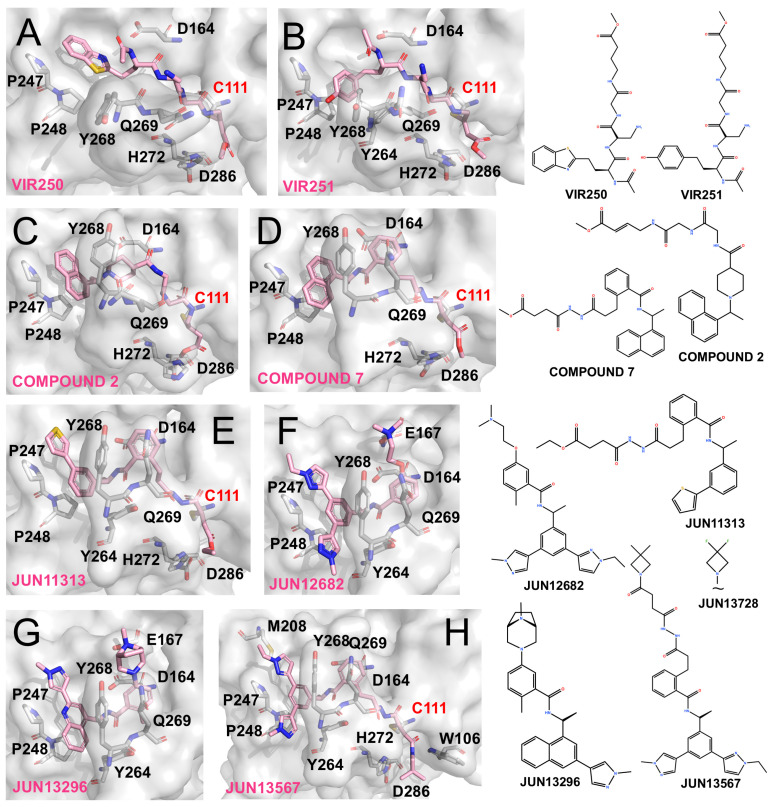
PLpro inhibitors based on VIR-derived and hybrid scaffolds (The surface and carbon atoms of PLpro are shown in gray and ligands in pink). (**A**,**B**) Structures of VIR250 and VIR251 bound to PLpro (PDB codes: 6WUU, 6WX4). (**C**) Compound **2** (PDB code: 8IHO). (**D**) Compound **7** (PDB code: 8EUA). (**E**,**F**) Covalent inhibitor Jun11313 and non-covalent inhibitor Jun12682 (PDB code: 8UVM, 8UOB). (**G**) Non-covalent compound JUN13296 (PDB codes: 9DNU). (**H**) JUN13567 (PDB code: 9D2K).

VIR250 and VIR251 incorporate the canonical LXGG substrate-recognition motif to mimic natural cleavage sites, while introducing electrophilic warheads to enable covalent modification of Cys111 [74,75]. Their structural features are shown in Figure 4A,B. Differences in the P4 substituents account for subtle variations in binding: the Abu side chain of VIR250 is deeply inserted into the P4 pocket, whereas the hTyr moiety of VIR251 is oriented toward the adjacent loop region. Consequently, the C-terminal segment of VIR250, together with its Dap side chains, packs closer to this loop than in VIR251. In contrast, downstream binding to the Gly-Gly motif remains largely conserved between them.

Both inhibitors mimic the LXGG substrate sequence and rely on backbone amino and carbonyl groups to form extensive ionic interactions with Gly163, Asp164, and loop backbone residues. Importantly, the vinyl methyl ester at the P1 position undergoes Michael addition, forming a covalent thioether linkage with Cys111 and thereby irreversibly blocking PLpro activity. Previous reports have shown that both compounds selectively and potently inhibit SARS-CoV and SARS-CoV-2 PLpro, with weaker activity against MERS-CoV and minimal effects on human deubiquitinases [74].

#### 4.3.2. Hybrid Designs Combining VIR and GRL0617 Scaffolds

The covalent engagement of Cys111 by VIR compounds has proven to be an effective strategy, while the high affinity of GRL0617-based scaffolds for the P3/P4 subsites suggests complementary opportunities. This observation has motivated the development of hybrid inhibitors that combine electrophilic warheads with GRL-derived backbones.

Inspired by the alkenyl warheads of VIR inhibitors, Wang et al. integrated this feature with Compound **12** (originally reported by Shan et al. [65]) to generate a covalent inhibitor Compound **2** (Figure 4C). Unlike Compound **12**, this hybrid adopts a distinct binding conformation at the P3/P4 region, with its naphthyl group failing to fully occupy the P4 pocket. Nevertheless, its vinyl warhead successfully engages Cys111, resulting in covalent inhibition [76]. Similarly, Sanders et al. designed Compound **7**, a GRL0617-inspired molecule bearing an appended vinyl electrophile (Figure 4D). Crystallographic analysis confirmed that this design retained the GRL-like binding mode at the P3/P4 subsites while enabling covalent modification of Cys111, yielding potent antiviral activity (EC_50_ ~1.1 μM) [77].

Building on insights from the BL2 groove recognition, Tan et al. emphasized the importance of the thiophene moiety in XR-8-24 and designed JUN11313 by combining this feature with a maleimide electrophile derived from Compound **7**. Co-crystal structures confirmed the covalent modification of Cys111 (Figure 4E), although the thiophene unexpectedly oriented toward the ubiquitin Val70-binding site, which they designated as the “Val70UB” pocket. Based on this discovery, the same group developed the non-covalent inhibitor JUN12682 (EC_50_ ~0.42 μM), which simultaneously engages multiple subsites, including the Val70UB site, the BL2 groove, and Glu167 within the BL2 loop. JUN12682 also demonstrated robust antiviral efficacy in both cell-based assays and animal models (Figure 4F) [71].

Further optimization of this scaffold yielded JUN13296 (IC_50_ 0.27 μM, EC_50_ 0.18 μM) and JUN13728. The co-crystal structure of JUN13296 reveals dominant interactions with the P4 hydrophobic channel, the Val70 site, and Glu167, consistent with its classification as a potent non-covalent inhibitor (Figure 4G). JUN13728 retains an extended side chain that enables covalent engagement of Cys111, classifying it as a covalent inhibitor [78]. The co-crystal structure of JUN13567, a close analogue of JUN13728, shows that the compound adopts a binding mode in which the tail region resembles that of JUN12682, engaging hydrophobic grooves and the Val70 site. Meanwhile, the elongated head extends toward and covalently reacts with Cys111 (Figure 4H) [79].

#### 4.3.3. Other Peptide-Based Inhibitors

Several additional peptide-based inhibitors have been reported, although detailed structural information remains limited. Protić et al. described the antimicrobial peptide gramicidin D, which inhibited PLpro with an IC_50_ of ~2.5 μM [80]. Liu et al. designed peptide-drug conjugates (PDCs) by linking GRL0617 to sulfonium-tethered peptides derived from the PLpro-specific LRGG motif. The resulting compounds, EM-C and EC-M, inhibited PLpro with IC_50_ values of 7.40 and 8.63 μM, respectively [81,82].

#### 4.3.4. Future Directions for Peptide Inhibitors

The first-generation peptide-derived VIR inhibitors demonstrated that covalent targeting of Cys111 is achievable despite the recessed and sterically constrained architecture of the PLpro catalytic cleft. By mimicking the canonical LXGG recognition motif and positioning an electrophilic warhead for Michael addition to Cys111, these compounds provided a clear proof-of-concept for covalent PLpro inhibition. Building on this foundation, subsequent efforts moved beyond purely peptide-like designs. They integrated covalent warheads with small-molecule elements that better complement the P3/P4 pocket, including GRL0617-inspired frameworks. This hybridization strategy proved effective, as exemplified by compounds like JUN11313 and its later derivatives. These compounds further exploit auxiliary subsites, such as the BL2 groove and the Val70 pocket, culminating in more optimized designs like JUN13567. Despite these structural successes, covalent inhibitors have generally lagged behind non-covalent small molecules in cellular and in vivo settings. This gap is likely due to the well-known limitations of peptide-like compounds, including poor membrane permeability, rapid metabolic clearance, and suboptimal systemic exposure, which can diminish antiviral efficacy even when target engagement is strong in biochemical assays.

Future development of peptide-based PLpro inhibitors will likely benefit from three complementary directions. First, peptidomimetic optimization, such as incorporation of non-natural amino acids, backbone cyclization, and rational scaffold simplification, could improve proteolytic stability and cellular uptake. Second, structure-guided hybrid design can integrate peptide- or warhead-derived elements into drug-like small-molecule cores, using GRL0617-like scaffolds for strong P3/P4 anchoring while maintaining covalent engagement. Third, delivery strategies like prodrugs, nanoparticles, or cell-penetrating motifs can improve exposure, bioavailability, and the translational potential of peptide-based inhibitors.

### 4.4. Other Structurally Characterized Small-Molecule Inhibitors—Diverse Scaffolds

**Figure 5:**
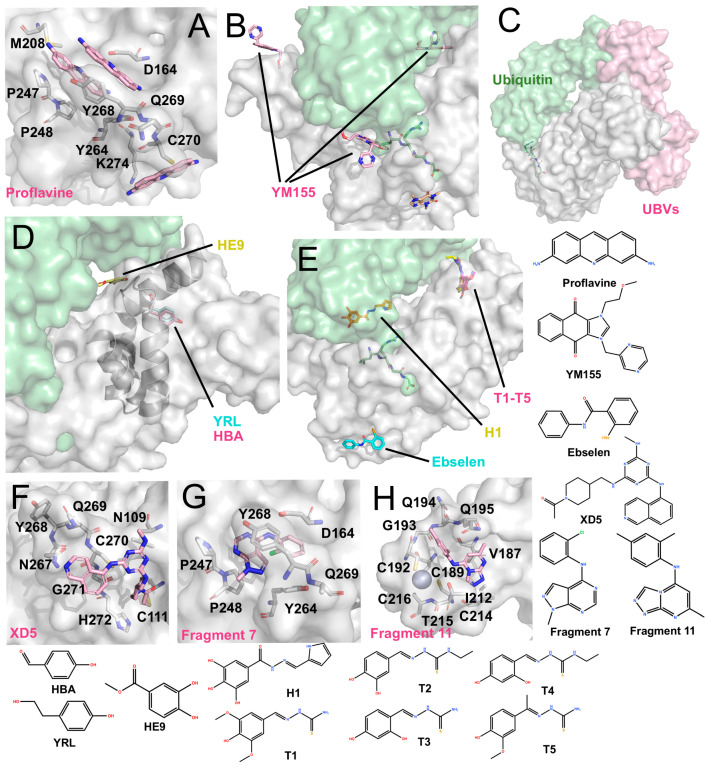
Other structurally characterized inhibitors (The surface and carbon atoms of PLpro are shown in gray, ubiquitin in green and ligands in pink, pile yellow or cyan). (**A**) Proflavine molecules bound near the active site (PDB code: 7NT4). (**B**) YM155 binding at multiple sites, including the active site, zinc-finger, and the S2 region (PDB code: 7D7L). ISG15 is shown in green. (**C**) Ubv (PDB code: 8CX9). (**D**) Phenolic inhibitors: YRL (PDB: 7OFS) and HBA (PDB code: 7OFT) bind between the Ubl and Thumb domains, while HE9 binds near the S2 site (PDB code: 7OFU). (**E**) Hydrazones and thiosemicarbazones: H1 binds near the S1 site, while T1–T5 bind between the Thumb and Ubl domains (PDB codes: 7QCG-7QCM). Ebselen binds near His272 at the base of the Palm domain (PDB code: 7M1Y). (**F**) XD5 covalently modifies Cys111, with the remaining moiety positioned on the exterior of the BL2 loop (PDB code: 8Z4W). (**G**) Fragment 7 binds near the P4 site (PDB code: 9BRW). (**H**) Fragment 11 binds near the zinc-finger domain (PDB code: 9BRX).

Several structurally diverse PLpro inhibitors have been identified through drug-repurposing screens and rational design and are presented here as representative examples with available structural data (Figure 5).

#### 4.4.1. Acriflavine (ACF)

ACF has been reported as a mixture of trypaflavines and proflavine with established antiviral and antimalarial activities [83,84,85], and as a potent PLpro inhibitor through a drug-repurposing screen. ACF exhibited IC_50_ values of ~1.17–1.46 μM against PLpro and potently suppressed SARS-CoV-2 replication in cells (EC_50_ ~64–84 nM) [86]. The co-crystal structure revealed that three ACF molecules occupy distinct positions: two within the P3-P4 subsites and one adjacent to the catalytic cleft (Figure 5A). The latter molecule forms parallel π-π stacking with His272 and T-shaped stacking interactions with Trp106. Within the P3-P4 pocket, the two molecules stack with each other and interact with Pro247, Tyr273, and Gly163 via polar contacts. Importantly, their binding induces a vertical reorientation of Tyr268, driving closure of the BL2 loop over the active site, thereby effectively occluding substrate access.

#### 4.4.2. YM155

Zhao et al. identified YM155, cryptotanshinone, and tanshinone I as PLpro inhibitors through a drug-repurposing screen, with YM155 showing the strongest activity (IC_50_ ~2.47 μM; EC_50_ ~0.17 μM) [87]. The co-crystal structure (Figure 5B) revealed three YM155 molecules bound per PLpro: at the P3-P4 active site, the zinc-finger domain, and the thumb domain. At the active site, YM155 mimics GRL0617 binding, occupying the P4 subsite and inducing Tyr268 to fold inward, thereby sealing the P3 pocket. At the zinc-finger site, YM155 inserts into the zinc-binding cleft, perturbing the coordinating loop without displacing zinc ions. At the thumb domain, YM155 engages in π-π stacking with Phe69, altering the S2 site critical for ubiquitin and ISG15 recognition. These interactions suggest that YM155 inhibits both the catalytic activity and deubiquitinating functions of PLpro, although further enzymatic validation is needed.

#### 4.4.3. Hydrazones and Thiosemicarbazones

Ewert et al. rationally designed hydrazone and thiosemicarbazone derivatives that selectively inhibit PLpro’s deubiquitinase and deISGylating activities [88]. Co-crystal structures revealed that hydrazone compound H1 binds near the *α*-helix adjacent to the S1 site, forming polar contacts with Gln174, Met206, Arg166, and Ser170. Additionally, it induces π–π stacking with Tyr171, a residue crucial for ubiquitin/ISG15 recognition. Thiosemicarbazone derivatives (e.g., T1–T5) were found to bind near the S2 thumb domain, engaging Arg65 and loop residues Asp76-Leu80 (Figure 5E). These interactions are proposed to sterically hinder ubiquitin and ISG15 binding. While in vitro assays confirmed inhibition of ubiquitin/ISG15 cleavage, quantitative IC_50_ values and cellular antiviral data were not reported.

#### 4.4.4. Ebselen and Ebselen-Derived Inhibitor—Prototype of Metal-Based Compounds

Ebselen was initially reported as a metal-binding inhibitor of Mpro, identified through drug-repurposing screens [15,89]. It is an organoselenium compound originally devel-oped as an antioxidant and anti-inflammatory agent. Structural studies revealed that it covalently modifies the catalytic Cys145 of Mpro via its selenium atom, although electron density for the remaining molecular framework was unresolved [90]. It’s in-hibitory activity has also been described as non-specific, as ebselen can inhibit a broad spectrum of cysteine proteases, including PLpro (IC50 ~10.3 μM) [91]. In addition, eb-selen is proposed to act as a zinc-ejecting agent, where its selenium atom disrupts Zn2+ binding and destabilizes the enzyme’s structure [92]. 

Osipiuk et al. reported the co-crystal structure of ebselen with a C111S PLpro mu-tant (Figure 5E, PDB code: 7M1Y, unpublished). Ebselen binds near Cys284 rather than Cys111, and its N-Se bond is cleaved. As a result, it remains unclear whether ebselen directly interacts with Cys111 to inhibit PLpro. Furthermore, Liu et al. designed several ebselen-based derivatives, including the covalent inhibitor 6c, which binds to Cys189 within the PLpro zinc-finger and inhibited enzyme activity by ~42.9% at 50 nM, with an EC_50_ of ~3.9 μM. They also identified a non-covalent analogue, 7e, that binds near the zinc-finger region and exhibits potent inhibition (IC50 ~63.1 nM; EC50 ~7.4 μM) [60]. Independently, Zmudzinski et al. evaluated multiple ebselen analogues against SARS-CoV-2 enzymes, identifying Compound **7** as the most potent PLpro inhibitor (IC50 ~0.58 μM), although corresponding cell-based antiviral data were not reported [93].

#### 4.4.5. Phenolic Compounds

Srinivasan et al. investigated phenolic derivatives (YRL, HBA and HE9) as PLpro inhibitors through screening of natural product-derived compound libraries [94]. Among these, HBA (IC_50_ ~3.99 μM) showed no cellular antiviral activity, whereas YRL (IC_50_ ~6.68 μM, EC_50_ ~1 μM) and HE9 (IC_50_ ~3.76 μM, EC_50_ ~0.13 μM) demonstrated antiviral efficacy. Structural studies (Figure 5D) showed that YRL and HBA bind within the cleft between the thumb and Ubl domains, altering the α-helix near the S2 site, potentially interfering with ubiquitin/ISG15 recognition. HE9 was observed to stack with His73 outside the S2 site, repositioning Glu69 and Phe70 to partially occupy the substrate-binding pocket. These findings underscore that natural product-derived compounds can act as S2 allosteric modulators of PLpro.

#### 4.4.6. Covalent Compound XD-5

Wang et al. obtained the compound XD-5 (IC_50_ ~1.3 μM) through high-throughput screening, which has a structure similar to modified GRL0617 analogues [95]. However, its co-crystal structure shows that the carbonyl head unexpectedly binds to Cys111 (Figure 5F), making it a covalent inhibitor, while most of the other moieties bind to the exterior of the BL2 loop. This observation suggests that access to Cys111 from this trajectory remains rare, and XD-5 may therefore represent a notable example of successfully targeting the buried catalytic cysteine.

#### 4.4.7. Fragment 7 and 11

Taylor et al. used an NMR-based fragment screening approach to identify several PLpro-binding fragments, among which fragments 5, 7, and 11 were confirmed through crystallography [73]. Fragment 5 has already been introduced earlier in the text as a representative fragment that binds to the Val70 site. Fragment 7 relies on T-shaped stacking with P248 and parallel π–π stacking with Y268 (Figure 5G). Although these binding sites partially inhibit PLpro activity, the overall inhibition is limited, with IC_50_ values in the millimolar range. Fragment 11 binds to a unique hydrophobic channel near the zinc ion, a previously unreported binding site. The structure shows that the zinc ion remains in situ at the fingertip, with no significant changes to the protein structure (Figure 5H). Accordingly, even at a concentration of 1 mM, Fragment 11 does not significantly inhibit PLpro activity, displaying only moderate binding affinity.

### 4.5. Protein Inhibitor: Ubvs

Compared with the extensive efforts devoted to small-molecule discovery, protein-based inhibition of PLpro remains at an earlier stage. A notable advance involved using phage display to engineer ubiquitin variants (Ubvs) that specifically target SARS-CoV-2 PLpro [96]. These Ubvs were found to inhibit PLpro enzymatic activity and reduce viral replication in Vero cells, offering early evidence that engineered protein scaffolds can act as antiviral modulators.

The co-crystal structure of PLpro bound to a Ubv revealed an unexpected binding mode. Rather than occupying the canonical S1–S2 interface engaged by natural ubiquitin, the Ubv dimer preferentially contacts the S2 site on the thumb domain. It interacts with multiple residues in the S2-region α-helix (including Asp62), creating steric hindrance that interferes with ubiquitin binding (Figure 5C). Consistent with this binding geometry, ubiquitin hydrolysis was strongly impaired, whereas ISG15 processing was less affected, leading to a substrate bias that preferentially suppresses deubiquitination over deISGylation. This selectivity contrasts with many small-molecule inhibitors, which often reduce both activities through competitive blockade near the catalytic cleft [96].

Recent structural studies of DMVs raise an important contextual consideration. Ubvs bind the S2 site and extend toward the “dorsal” surface of the PLpro right-hand fold. However, this face may be partially occluded in the DMV-embedded nsp3 architecture. Despite this, Ubvs still inhibit viral replication in cells. One possibility is that Ubv binding perturbs PLpro-mediated interactions within nsp3, indirectly affecting DMV organization or function, although this remains to be experimentally validated. These findings suggest that PLpro is not only a protease but also a structural and regulatory element within the nsp3-containing replication organelle. Accordingly, targeting PLpro to disrupt DMV-associated functions could provide an additional antiviral strategy that complements direct catalytic inhibition.

Despite their mechanistic appeal, Ubvs face the well-known constraints of protein therapeutics, including issues with stability, delivery, and potential immunogenicity. As a result, Ubvs have been presented primarily as proof-of-concept tools, rather than near-term clinical candidates. Nonetheless, by engaging noncanonical surfaces that are difficult for small molecules to access, engineered protein scaffolds expand the addressable interaction space on PLpro. This illustrates how high selectivity can be achieved through protein engineering, while highlighting the practical challenges that must be overcome for therapeutic translation.

### 4.6. Inhibitors Reported Without Structural Validation

Several molecules have been reported to inhibit PLpro, but they lack direct structural validation. We briefly summarize representative compounds, with their chemical structures, IC_50_ and EC_50_ values provided in Supplementary Table S2.

#### 4.6.1. Repurposed or Clinical Agents

Olmutinib, an EGFR inhibitor identified through high-throughput screening, dis-played strong enzymatic inhibition (IC_50_ 0.54 μM) and cellular antiviral activity (EC_50_ 9.76 μM). However, its narrow therapeutic window (CC_50_ ~12.5 μM) limited its further development [97]. Tropifexor, a farnesoid X receptor agonist currently in phase II clin-ical trials, inhibited PLpro (IC_50_ ~5.11 μM) and reduced viral replication (EC_50_ ~10.6 μM) [98,99]. The purine analogue 6-thioguanine (6-TG), inhibited PLpro from SARS-CoV, MERS-CoV, and SARS-CoV-2, with an EC_50_ of ~2.13 μM against SARS-CoV-2 replication, representing one of the few compounds with apparent broad-spectrum potential [100,101,102,103]. By contrast, cetylpyridinium chloride, a quater-nary ammonium surfactant, inhibited PLpro with an IC_50_ of ~2.72 μM [97], though its detergent-like properties raise concerns about nonspecific effects.

#### 4.6.2. Covalent Inhibitors

Representative examples include Compound **1**, which carries a chlorine-based electrophile and was predicted to bind near the active site with π-π stacking prior to covalently modifying Cys111 (IC_50_ ~18 μM) [104]. Another example is LY1, designed using a pharmacophore-based approach, which likely undergoes Michael addition with Cys111 and shows antiviral activity in cells (EC_50_ ~3.9 μM) [105]. Oridonin, a natural diterpenoid containing an α,β-unsaturated ketone, suppresses viral replication (EC_50_ ~1.85 μM) despite weak enzymatic inhibition (IC_50_ ~600 μM) [106]. RI173, a thiuram disulfide derivative, exhibits strong enzymatic potency (IC_50_ ~0.2 μM) but ex-treme cytotoxicity likely driven by nonspecific reactivity (CC_50_ ~0.3 nM) [107]. These compounds may serve as valuable leads to address the current lack of PLpro inhibitors.

#### 4.6.3. Natural Product-Derived Aromatics

Reported examples include flavonoids such as chrysin 7-O-*β*-D-glucuronide, which inhibited SARS-CoV-2 PLpro with IC_50_ ~2.54 μM and reduced viral replication (EC_50_ ~8.72 μM) [108], and IXN, a hop-derived compound with only moderate potency (IC_50_ ~59 μM; EC_50_ ~20.1 μM) [109]. Among phenolic compounds, 1,2,3,4,6-penta-O-galloylglucose (PGG) inhibited SARS-CoV-2 PLpro (IC_50_ ~3.9 μM) but showed pronounced cytotoxicity (CC_50_ ~7.7 μM) [110]; aurintricarboxylic acid displayed weaker inhibition (IC_50_ ~30 μM; EC_50_ ~50 μM) [18]; and anacardic acid inhibited PLpro with IC_50_ ~24.3 μM, though antiviral activity was not assessed [111].

#### 4.6.4. Synthetic Heteroaromatic Scaffolds

Quinoline, pyrimidine, and indole derivatives have also been investigated as syn-thetic heteroaromatic scaffolds for SARS-CoV-2 PLpro inhibition. Among them, the 2-chloroquinoline analogue C10 showed potent enzymatic activity (IC_50_ ~0.35 μM) with low cytotoxicity (CC_50_ > 200 μM), although its antiviral efficacy has not yet been tested in cells [112]. A series of mercapto-pyrimidines yielded moderate inhibition, with Compound **5** (IC_50_ ~5.1 μM) and its optimized analogue 5E (IC_50_ ~0.85 μM) demonstrating improved potency, but showing only limited cellular antiviral effects [113]. Similarly, indole-based SIMR compounds provided promising leads: SIMR3030 was the most active enzymatic inhibitor (IC_50_ ~0.089 μM), though it translated into only modest antiviral efficacy in cell assays (EC_50_ ~27 μM) [114]. Other reported GRL0617-related analogues include F0213 (IC_50_ ~7.4 μM; EC_50_ ~4.5 μM) [115] and a pyridinyl-oxadiazole derivative (Compound **5**, IC_50_ ~7.19 μM) [116], though both re-main at the preliminary validation stage without supporting structural evidence. 

#### 4.6.5. Metal-Based Compounds

Zinc pyrithione inhibited SARS-CoV-2 PLpro with sub-micromolar potency (IC_50_ ~0.5 μM) and effectively suppressed SARS-CoV-2 replication in cells (EC_50_ ~0.84 μM) [57]. Maria Gil Mole et al. conducted a broader evaluation of transition-metal com-plexes and identified gold (Au-34) and silver (Ag-4b) derivatives as particularly active. Au-34 inhibited PLpro (IC_50_ ~0.09 μM) with reported antiviral efficacy and low cyto-toxicity. Ag-4b exhibited strong enzymatic inhibition (IC_50_ ~0.39 μM) and potent anti-viral activity (EC_50_ ~0.58 μM) [56,58].

## 5. Conclusions and Outlook

SARS-CoV-2 is the seventh coronavirus to infect humans and the third to cause a global pandemic [104]. The two earlier outbreaks were caused by SARS-CoV in 2003 and MERS-CoV in 2012 [105]. Historically, coronavirus drug discovery has centered on the RNA-dependent RNA polymerase (RdRp) and Mpro, but growing attention is now being directed toward PLpro. Beyond its essential role in viral polyprotein processing, PLpro antagonizes host innate immunity by removing ubiquitin and ISG15 modifications, thereby suppressing interferon signaling and contributing to inflammatory pathology. This dual role underscores its critical value as an antiviral target. Combination therapies that pair PLpro inhibitors with Mpro or RdRp inhibitors (e.g., nirmatrelvir or remdesivir) may not only enhance antiviral efficacy but also reduce the likelihood of resistance development.

The diverse classes of inhibitors described in this review reveal a spectrum of complementary strengths and limitations that collectively define the current landscape of PLpro-targeted drug discovery. Non-covalent small molecules generally offer the most favorable balance between structural tractability, selectivity, and translational potential, due to their tunable interactions with the P3/P4 region and adjacent auxiliary subsites. However, they face a significant challenge, as their enzymatic potency is often disconnected from antiviral activity at the cellular and animal levels, with biological efficacy still needing improvement. Covalent inhibitors, especially those from peptide-like scaffolds, offer clear target engagement and mechanistic clarity. However, they often encounter issues with cellular permeability, metabolic stability, and systemic exposure, limiting their in vivo effectiveness. Metal-based and zinc-finger–directed compounds further expand the accessible chemical space and reveal structural vulnerabilities of PLpro, but their broad reactivity toward cysteine proteases complicates selectivity and safety assessments. Natural products face challenges such as low activity and insufficient validation of their mechanisms and binding modes, requiring extensive further high-throughput screening. Protein-based binders, exemplified by ubiquitin variants, show that non-canonical and extended surfaces on PLpro can be potently and selectively targeted. However, their therapeutic applicability remains constrained by the inherent challenges of macromolecular drugs.

Enzymatic potency and crystallographically confirmed binding do not always predict therapeutic success. As seen with Mpro, some validated binders fail orthogonal assays or developability filters (e.g., nonspecific reactivity, aggregation, or weak cellular engagement) [117]. A similar approach should be applied to PLpro: prioritization must include IC_50_, orthogonal biochemical assays, cellular antiviral activity, cytotoxicity, counterscreens against host deubiquitinating enzymes, and, when possible, cellular target engagement. Chemotypes with consistent co-structures and robust cellular/animal phenotypes are more likely to translate effectively under these criteria.

With the continued integration of artificial intelligence into drug discovery, PLpro inhibitor development may increasingly benefit from AI-assisted design approaches. Such strategies have the potential to accelerate the identification of novel chemical motifs and previously unrecognized binding pockets, thereby further expanding the accessible chemical space. Recently, Insilico Medicine has reported notable progress with the AI-designed small-molecule drug renosertib for idiopathic pulmonary fibrosis [118]. The discovery and optimization of renosertib were driven by Insilico Medicine’s proprietary AI platform Pharma.AI, incorporating its generative molecular design engine, Chemistry42 [119]. Notably, PF-07957472 discussed in this review was also developed using AI-assisted design strategies [70], underscoring the potential of AI-driven approaches for the discovery of PLpro inhibitors.

More recently, advances in AI-guided protein design using tools such as RFdiffusion, BindCraft, and ProteinMPNN, have yielded synthetic binders with enhanced affinity and specificity [120,121,122], capable of accessing noncanonical sites that are difficult for small molecules to target. A study demonstrated the design of a miniprotein that in-hibits SARS-CoV-2 Mpro by targeting its dimerization interface [123]. Such approach-es will broaden the therapeutic landscape beyond classical small-molecule scaffolds and may help address the current gap in protein-based inhibitors targeting PLpro.

At the same time, more innovative treatments and drug delivery methods are emerging as highly promising research directions. For example, proteolysis targeting chimeras (PROTACs) technology has been attempted for the inhibition of Mpro [124], but the ubiquitin labeling required for this approach could be counteracted by PLpro. Therefore, adapting PROTAC strategies to directly target PLpro, or combining PROTAC approaches with PLpro inhibitors, may improve the feasibility of this modality. Similarly, molecular glue strategies have been proposed in the context of viral protease targeting [125], though none have yet been reported for PLpro. Extending such approaches to selectively destabilize PLpro or disrupt full-length nsp3 complexes represents a promising direction for next-generation antiviral design. Comparative studies of PLpro orthologs from MERS-CoV, HKU1, and other coronaviruses also highlight subtle differences in the S2 and ubiquitin-binding sites, offering structural clues for broad-spectrum inhibitor development.

In conclusion, while challenges remain, rapid progress in structural biology, medicinal chemistry, and computational approaches is transforming our understanding of SARS-CoV-2 PLpro and accelerating the pace of inhibitor discovery. With continued integration of experimental and AI-assisted strategies, the next few years are expected to deliver both deeper mechanistic insights and translationally relevant antiviral candidates.

## Data Availability

No new data were created or analyzed in this study.

## Supplementary Materials

The following supporting information can be downloaded at: https://www.mdpi.com/article/10.3390/molecules31030474/s1, Table S1: SARS-CoV-2 PLpro reported crystal structures; Table S2: Main SARS-CoV-2 PLpro inhibitors mentioned in this paper.
