# Supplementary material for: Structural Basis and Inhibitor Development of SARS-CoV-2 Papain-like Protease
Source: Molecules. 2026 Jan 29;31(3):474. doi: 10.3390/molecules31030474 (PMC12899064; doi:10.3390/molecules31030474)
Supplement: Supplementary file 1 [file molecules-31-00474-s001.zip › Appendix Table S1.pdf]

Table S1. SARS-CoV-2 PLpro reported crystal structures.

**PDB code**	**Protein**	**Ligand**	**Resolution /Å**	**Space group**	**Deposition authors or references**
6XA9	WT	ISG15 CTD	2.90	$P4_12_12$	[25]
6XAA	WT	Ubiquitin	2.70	$P2_12_12$	[25]
6YVA	C111S	ISG15	3.18	$P6_222$	[32]
6W9C	WT	-	2.70	*C*2	Osipiuk *et al.*
7RBR	C111S	Ubiquitin	1.88	*C*2	[52]
7RBS	C111S	ISG15	2.98	$C222_1$	[52]
7UV5	C111S-D286N	K48 linked di-ubiquitin	1.45	*C*2	[52]
7JRN	WT	GRL0617	2.48	$P2_1$	[64]
7CJM	C111S	GRL0617	3.20	$I4_122$	[59]
7CMD	WT	GRL0617	2.59	$P2_1$	[61]
7CJD	C111S	-	2.50	*C*2	[61]
6WZU	WT	-	1.79	$P3_221$	[62]
6WRH	C111S	-	1.60	$P3_221$	[62]
6XG3	C111S	-	2.48	$P3_221$	[62]
7JIR	C111S	GRL0617	2.09	$I4_122$	[62]
7JIT	C111S	Compound 2	1.95	$I4_122$	[62]
7JIV	C111S	Compound 3	2.05	$I4_122$	[62]
7JIW	WT	Compound 3	2.30	$I4_122$	[62]
7RZC	WT	JUN9-84-3	2.04	$P2_12_12_1$	Osipiuk *et al.* / [63]
7SQE	C111S	JUN9-84-3	2.00	$P2_12_12_1$	Osipiuk *et al.* / [63]
7SDR	WT	JUN9-72-2	2.72	$P2_12_12_1$	Osipiuk *et al.* / [63]
7JN2	WT	PLP_Snyder441	1.93	$I4_122$	Osipiuk *et al.*
7KRX	C111S	PLP_Snyder441	2.72	$I4_122$	Osipiuk *et al.*
7KOJ	C111S	PLP_Snyder494	2.02	$I4_122$	Osipiuk *et al.*
7KOL	WT	PLP_Snyder496	2.58	$I4_122$	Osipiuk *et al.*
7KOK	C111S	PLP_Snyder496	2.00	$I4_122$	Osipiuk *et al.*
7SGU	C111S	PLP_Snyder608	1.79	$P3_221$	Osipiuk *et al.*
7SGV	C111S	PLP_Snyder630	2.00	$P3_221$	Osipiuk *et al.*
7SGW	WT	PLP_Snyder630	1.95	$P3_221$	Osipiuk *et al.*
8G62	C111S	Remodilin NCGC 390004	2.17	$P4_12_12$	Osipiuk *et al.*

7D6H	C111S	-	1.60	$P3_{2}21$	[65]
7E35	C111S	Compound 12	2.40	$P6_{5}22$	[65]
7TZJ	C111S	3k	2.66	$P2_{1}2_{1}2$	[66]
7LBR	WT	XR8-89	2.20	$P2_{1}$	[67]
7LBS	WT	XR8-24	2.80	$P2_{1}$	[67]
7LLF	WT	XR8-83	2.30	$P2_{1}$	[67]
7LLZ	WT	XR8-69	2.90	$P2_{1}$	[67]

Table S1 (continued)

**PDB code**	**Protein**	**Ligand**	**Resolution /Å**	**Space group**	**Deposition authors or references**
7LOS	WT	XR8-65	2.90	$P2_{1}$	[67]
6WUU	WT	VIR250	2.79	$P2_{1}$	[74]
6WX4	WT	VIR251	1.66	$I222$	[74]
8IHO	WT	Compound 2	2.55	$P2_{1}2_{1}2_{1}$	[76]
8EUA	WT	Compound 7	3.10	$I4_{1}22$	[77]
8UVM	WT	JUN11313	2.52	$I4_{1}22$	[71]
8UUF	WT	JUN11941	2.84	$I4_{1}22$	[71]
8UUG	WT	JUN12303	2.74	$I4_{1}22$	[71]
8UUH	WT	JUN12199	2.80	$I4_{1}22$	[71]
8UUU	WT	JUN12162	3.01	$I4_{1}22$	[71]
8UUV	WT	JUN12197	3.01	$I4_{1}22$	[71]
8UUW	WT	JUN12145	3.20	$I4_{1}22$	[71]
8UUY	WT	JUN12129	3.05	$I4_{1}22$	[71]
8UOB	WT	JUN12682	2.54	$I4_{1}22$	[71]
7M1Y	C111S	Ebselen	2.02	$P6_{5}22$	Osipiuk *et al.*
7NT4	WT	ACF	2.68	$P6_{5}22$	[86]
7D7K	C111S	-	1.90	$P6_{5}22$	[87]
7D7L	C111S	YM155	2.11	$P6_{5}22$	[87]
7QCG	C111CSO	H1	1.75	$P3_{2}21$	[88]
7QCH	C111CSO	T1	1.88	$P3_{2}21$	[88]
7QCI	C111CSO	T2	1.76	$P3_{2}21$	[88]
7QCJ	C111CSO	T4	1.84	$P3_{2}21$	[88]
7QCK	C111CSO	T3	1.92	$P3_{2}21$	[88]
7QCM	C111CSO	T5	1.77	$P3_{2}21$	[88]
7NFV	WT	-	1.42	$P3_{2}21$	[94]
7OFS	WT	YRL	1.90	$P3_{2}21$	[94]
7OFT	WT	HBA	1.95	$P3_{2}21$	[94]
7OFU	WT	HE9	1.72	$P3_{2}21$	[94]
8CX9	C111S	Ubv	3.50	$P2_{1}$	[96]
7D47	C111S	-	1.97	$P3$	Wu *et al.*
7YBG	C111S	-	1.90	$P3_{2}21$	Zeng *et al.*
8FWN	C111S	-	1.50	$P3_{2}21$	Bezerra *et al.*

3E9S	SARS-CoV PLpro	GRL0617	2.50	$I222$	Mesecar *et al.*
9BF7	C111S	-	1.68	$P3_{2}21$	[12]
9BF8	WT		1.85	$P3_{2}21$	[12]
9VWY	C270S	-	1.83	$P3_{2}21$	Arya *et al.*
9VAO	WT	-	1.82	$P3_{2}21$	Arya *et al.*
8XTD	C111S	Linagliptin	2.70	$P3_{2}21$	Choudhary *et al.*

Table S1 (continued)

**PDB code**	**Protein**	**Ligand**	**Resolution /Å**	**Space group**	**Deposition Authors or references**
8X1X	C111S	Lithocholic acid	2.30	$P3_{2}21$	Choudhary *et al.*
8ZSE	C111S	GZNL-2002	2.80	$P4_{1}22$	Lu *et al.*
9F7P	C112S	-	1.70	$P3_{2}21$	Camara-Artigas *et al.*
9F7Q	C112S	-	2.30	$P3_{2}21$	Camara-Artigas *et al.*
9F7R	C112S	-	1.50	$P3_{2}21$	Camara-Artigas *et al.*
9F7S	C112S-K191D-K229R	-	1.80	$P3_{2}21$	Camara-Artigas *et al.*
9F7T	C112S-K191D-K229R	-	2.05	$I4_{1}22$	Camara-Artigas *et al.*
9F7U	C112S-K191D-Q222D-K229R-Q230R-C271S	-	1.60	$P3_{2}21$	Camara-Artigas *et al.*
9F7Y	C112S-K191D-Q222D-K229R-Q230R-C271S	-	1.80	$P3_{2}21$	Camara-Artigas *et al.*
9CYB	C111S	WEHI-P1	1.98	$P22_{1}2_{1}$	[72]
9CYC	C111S	WEHI-P2	2.01	$P22_{1}2_{1}$	[72]
9CYD	C111S	WEHI-P4	2.80	$P4_{3}32$	[72]
9CYK	C111S	WEHI-P24	1.88	$P22_{1}2_{1}$	[72]
9D2K	WT	JUN13567	2.70	$P4_{1}2_{1}2$	[78]
8Z4W	WT	XD-5	2.33	$P1$	[95]
9DNU	WT	JUN13296	2.30	$I4_{1}22$	[79]
9DNV	WT	JUN13308	2.40	$I4_{1}22$	[79]
9DO1	WT	JUN13307	2.40	$I4_{1}22$	[79]
9DO3	WT	JUN13317	2.50	$I4_{1}22$	[79]
9DO5	WT	JUN12265	3.00	$I4_{1}22$	[79]
9DOI	WT	JUN13306	2.30	$I4_{1}22$	[79]

8YX2	C111S	GZNL-P4	2.31	$P2_12_12_1$	[69]
8YX3	C111S	GZNL-P28	2.60	$P2_12_12_1$	[69]
8YX4	C111S	GZNL-P31	2.28	$I4_122$	[69]
8YX5	C111S	GZNL-P35	1.74	$P2_1$	[69]
9CSY	C111S	PF-07957472	2.60	$P2_12_12_1$	[70]
9BRV	C111S	Fragment 5	2.60	$P3_2$	[73]
9BRW	C111S	Fragment 7	2.50	$P2_12_12_1$	[73]
9BRX	C111S	Fragment 11	1.80	$P3_221$	[73]
8JUX	C111S	SR-01	3.20	$P6_522$	Hsieh *et al.*
