# Supplementary material for: Structural Basis and Inhibitor Development of SARS-CoV-2 Papain-like Protease
Source: Molecules. 2026 Jan 29;31(3):474. doi: 10.3390/molecules31030474 (PMC12899064; doi:10.3390/molecules31030474)
Supplement: Supplementary file 1 [file molecules-31-00474-s001.zip › Appendix Table S2.pdf]

Table S2. Main SARS-CoV-2 PLpro inhibitors mentioned in this paper.

**Name**	**Structure**	**IC_{50}/μM**	**EC_{50}/μM**	**Comments**	**References**
GRL0617	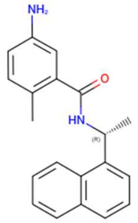	2.1	1.4	SARS-CoV PLpro inhibitor	[59–62]
Compound 2	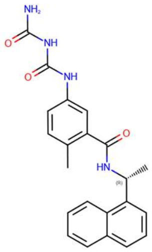	5.1	-	-	[62]
Compound 3	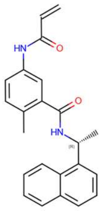	6.4	-	-	[62]
PLP_Snyder494	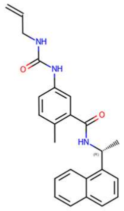	-	-	-	Osipiuk *et al.*
PLP_Snyder496	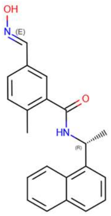	-	-	-	Osipiuk *et al.*
PLP_Snyder608	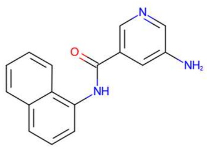	-	-	-	Osipiuk *et al.*

Table S2 (continued)

Name	Structure	IC_{50}/μM	EC_{50}/μM	Comments	References
PLP_Snyder630	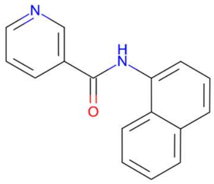	-	-	-	Osipiuk *et al.*
Jun9-84-3	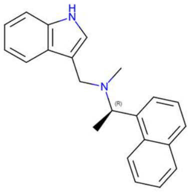	0.67	17.07	-	[63]
Jun9-72-2	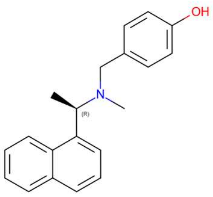	0.67	7.93	-	[63]
Compound 12	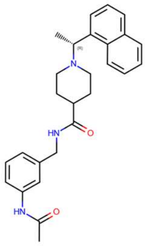	2.00	-	-	[65]
Compound 3k	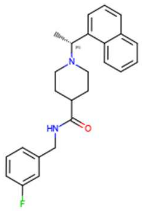	1.50	-	-	[66]

XR-8-24	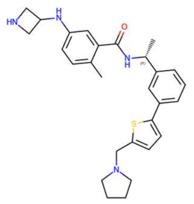	0.56	<2	-	[67]

Table S2 (continued)

**Name**	**Structure**	**IC50/μM**	**EC50/μM**	**Comments**	**References**
Compound 42	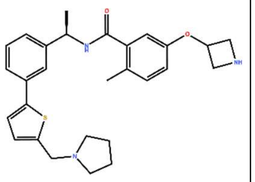	0.81		-	[68]
Compound 10 (XR-8-23)	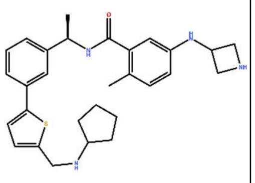	0.39		-	[68]
GZNL-P35	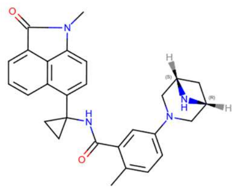	0.008	-	-	[69]
PF-07957472	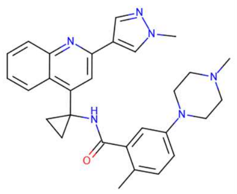	-	0.147	-	[70]
VIR250	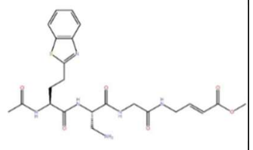	Covalent	-	-	[74]
VIR251	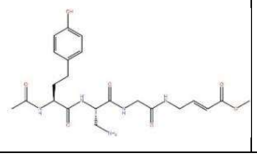	Covalent	-	-	[74]

Compound 2	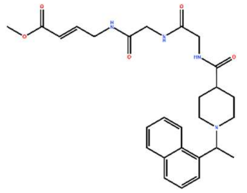	Covalent	-	-	[76]
Compound 7	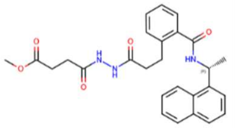	Covalent	1.1	-	[77]

Table S2 (continued)

**Name**	**Structure**	**IC_{50}/μM**	**EC_{50}/μM**	**Comments**	**References**
JUN11313	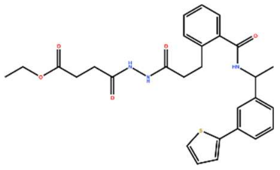	Covalent	-	-	[71]
JUN12682	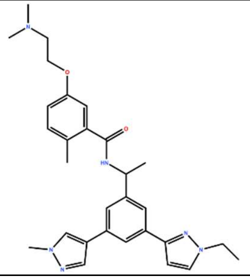	Covalent	-	-	[71]
JUN13296	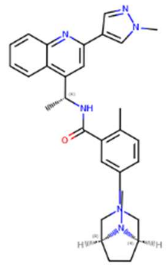	0.27	0.18	-	[78]
JUN13567	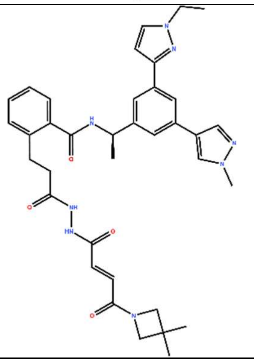	Covalent	-	-	[79]
JUN13728	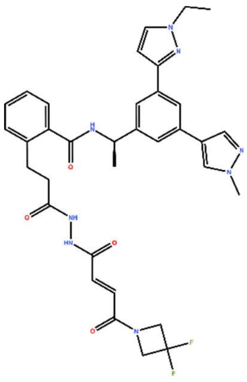	Covalent	0.35	-	[79]
Gramicidin D	Peptide	2.5	-	-	[80]

EM-C	PDC	7.40	-	-	[81]
EC-M	PDC	8.63	-	-	[81]

Table S2 (continued)

Name	Structure	IC_{50}/μM	EC_{50}/μM	Comments	References
WEHI-P4	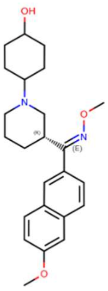	0.019	-	-	[72]
Proflavine	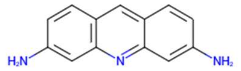	1.17 -1.46	0.064 -0.084	-	[86]
YM155	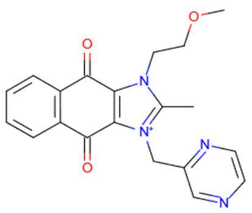	2.47	0.17	-	[87]
H1	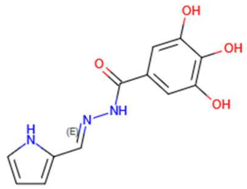	-	-	-	[88]
T1	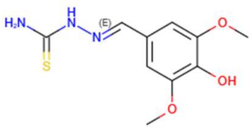	-	-	-	[88]
HBA	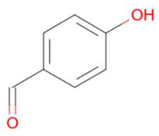	3.99	-	-	[94]

YRL	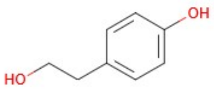	6.68	1	-	[94]

Table S2 (continued)

**Name**	**Structure**	**IC_{50}/μM**	**EC_{50}/μM**	**Comments**	**References**
HE9	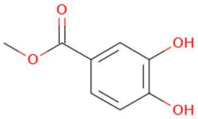	3.76	0.13	-	[94]
XD-5	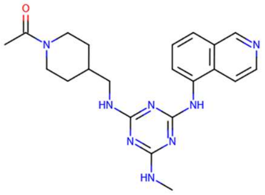	1.3	-	-	[95]
Fragment 5	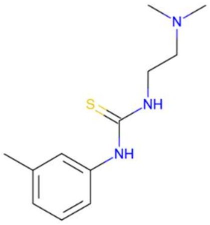	-	-	-	[73]
Fragment 7	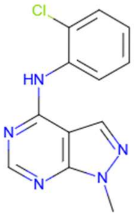	-	-	-	[73]
Fragment 11	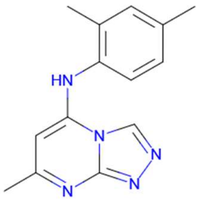	-	-	-	[73]

6c	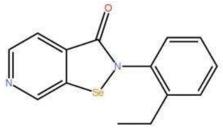	-	3.9	42.9% Percent inhibition in 50 nm	[69]
7e	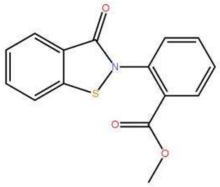	0.063	7.4	-	[69]

Table S2 (continued)

**Name**	**Structure**	**IC_{50}/μM**	**EC_{50}/μM**	**Comments**	**References**
7	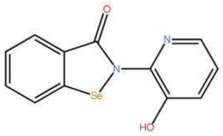	0.58	-	-	[60]
Chrysin-7-O-*β*-D-glucuronide	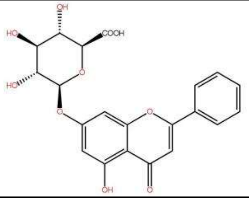	2.54	8.72	-	[108]
IXN	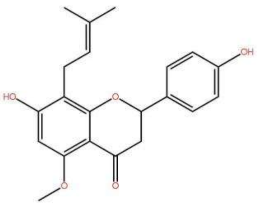	59	20.1	-	[109]
F0213	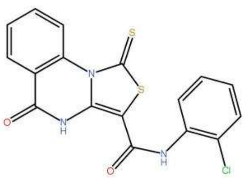	7.4	4.5	Inhibit virus in cells.	[115]
6-TG	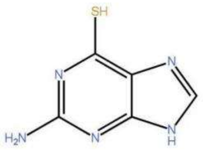	-	2.13	Cleavage assay by SDS-PAGE.	[103]

PGG	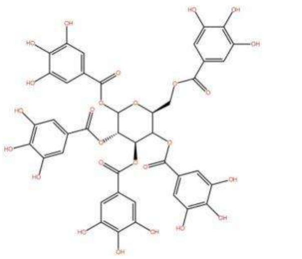	3.9	-	CC_{50} 7.7 μM, cytotoxicity.	[110]
Oridonin	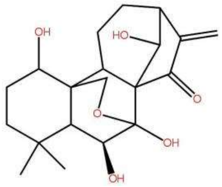	~600	1.85	-	[106]
SIMR3030	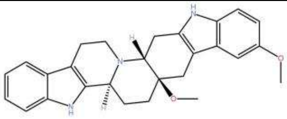	0.089	27	-	[114]

Table S2 (continued)

**Name**	**Structure**	**IC_{50}/μM**	**EC_{50}/μM**	**Comments**	**References**
5	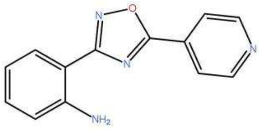	7.19	-	-	[116]
Aurintricarb-oxylic acid	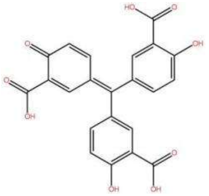	30	50	-	[18]
Anacardic acid	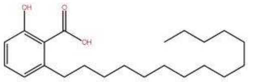	24.26 /11.45	-	-	[111]
Cetylpyridin-ium chloride	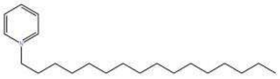	2.72	-	Mpro inhibitor.	[112]
Olmutinib	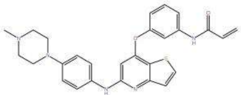	0.54	9.76	CC_{50} 12.48 μM, cytotoxicity.	[112]
C10	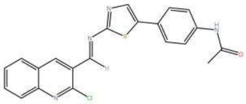	0.35	-	Mpro inhibitor.	[112]
Tropifexor	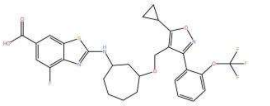	5.11	10.6	-	[99]

Compound 5	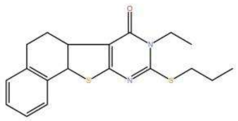	5.1	-	Inhibit virus in cells.	[116]
Compound 5E	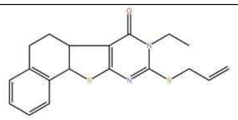	0.85	-	Can not inhibit virus in cell.	[116]
RI173	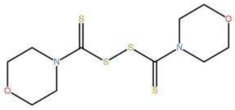	0.2	0.0001	Low targeting, cytotoxicity.	[107]
Compound 1	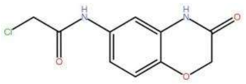	18	-	Covalent inhibitor.	[104]

Tabel S2 (continued)

Name	Structure	IC$_{50}$/μM	EC$_{50}$/μM	Comments	References
LY1	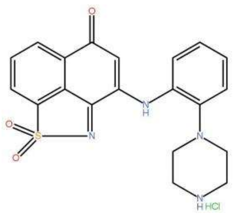	-	3.9	Covalent inhibitor, Mpro inhibitor.	[105]
Zinc pyrithione	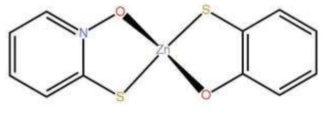	0.5	0.84	-	[57]
Au-34	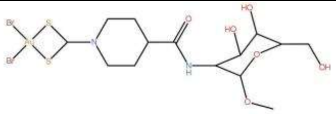	0.09	-	-	[56]
Ag-4b	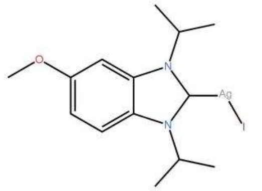	0.39	0.58	-	[58]
